# Patients with ≥ 20 × 10^9^/L Platelets at Baseline May Have a Prompt Response to Romiplostim During the Early Phase of Treatment: an Italian Single-Institution Experience

**DOI:** 10.4084/MJHID.2012.044

**Published:** 2012-06-28

**Authors:** Simone Baldini, Luigi Rigacci, Valentina Carrai, Renato Alterini, Rajmonda Fjerza, Alberto Bosi

**Affiliations:** University of Florence, Hematology Unit, Azienda Ospedaliero-Universitaria Careggi, Florence, Italy

## Abstract

Patients with chronic immune thrombocytopenia treated with romiplostim may benefit from a higher starting dose when a rapid increase in count is needed, but it could be avoided in those with a prompt response to the standard dosage. We hypothesized that a platelet count ≥ 20 × 10^9^/l at baseline could distinguish subjects with such response from those with a delayed one during the early phase of treatment. Our work is a retrospective and single-institution analysis comparing the median platelet count, the median weekly dosage of romiplostim and the median number of weekly platelet counts < 50 × 10^9^/l between patients with a baseline ≥ 20 × 10^9^/l platelets (n=10, 2 splenectomized) and those with a lower one (n=8, 3 splenectomized) during the first month of treatment with romiplostim. The results show a higher median platelet count (79,5 vs 40,5 × 10^9^/l, p=0,002) and a lower median dose of romiplostim (1 vs 2 mcg/kg/week, p=0,01) in subjects with a baseline ≥ 20 × 10^9^/l platelets, who also had a trend of less weekly counts < 50 × 10^9^/l platelets (1 vs 2, p=0,054). These data suggest that patients with ≥ 20 × 10^9^/l platelets at baseline may achieve a prompt response with the standard dose of romiplostim, but further and larger data are needed in order to assess whether it can be considered in clinical practice.

## Introduction

Romiplostim is an effective treatment for chronic immune thrombocytopenia (ITP) but time to response (platelet count ≥ 50 × 10^9^/l) is considerably variable during the early phase of treatment.^1^–^4^ Prompt responses are needed in particular clinical settings (bleedings, important comorbidities, anticoagulation) and the usual starting dose may be too small in these situations.^5^ We have explored in this retrospective study if 18 patients treated in our institution with a baseline platelet count (BPC) ≥ 20 × 10^9^/l might have a prompt response to the standard dose of romiplostim.

## Patients and Methods

We retrospectively analysed 18 consecutive chronic ITP patients treated with romiplostim in our institution up to July 2011. Romiplostim was prescribed as they had previous treatments failure and were unable to maintain a platelet count ≥ 30 × 10^9^/l without bleedings despite the ongoing treatment. One patient maintained a platelet count between 30–50 × 10^9^/l with steroids but was switched to romiplostim because of an uncontrolled diabetes mellitus. Among not splenectomized patients, 10/13 did not perform splenectomy because of clinical choice while 3/13 refused the intervention. Romiplostim was administered every 7±1 days starting from 1 mcg/kg and adjusting dosage on the basis of platelet count (< 50 × 10^9^/l: dose increased by 1 mcg/kg; 50–400 × 10^9^/l: dose maintained; > 200 × 10^9^/l for 2 consecutive weeks: dose reduced by 1 mcg/kg; > 400 × 10^9^/l: dose withheld and restarted at < 200 × 10^9^/l platelets reduced by 1 mcg/kg). BPC was assessed on the day of the first administration of romiplostim. All the patients were on steroid treatment (unchanged from at least 4 weeks) which was tapered as platelet count was ≥ 50 × 10^9^/l. Patients were divided in two groups on the basis of a BPC ≥ 20 × 10^9^/l or lower. Baseline characteristics and percentages of different previous treatments other than splenectomy among the two groups are summarised in tables 1 and 2 respectively. Response to romiplostim was evaluated in each group by the median value of weekly platelet count and by the median number of weekly platelet counts < 50 × 10^9^/l (both between weeks 2–5). Median dosage of romiplostim (weeks 1- 4) was also evaluated. Results were compared between the groups using the two tailed Mann-Whitney U test at a significance level of 0,05.

## Results

**Figure 1:**
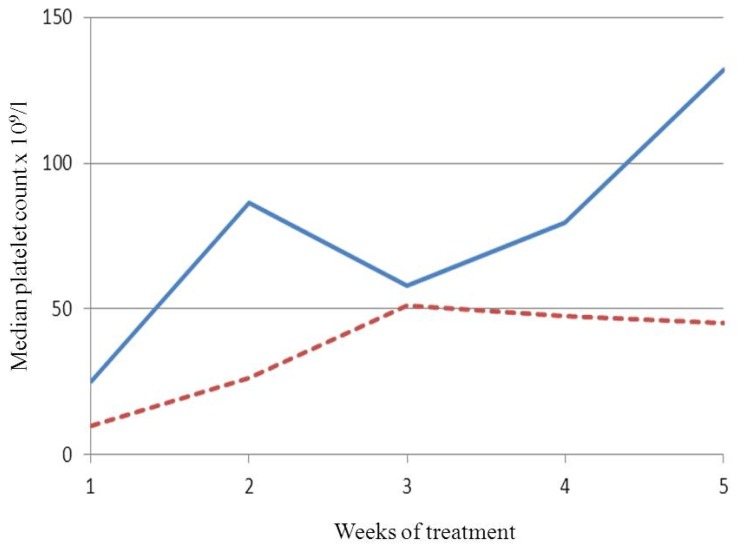
Median weekly platelet counts during the first month of treatment with romiplostim in patients with a baseline count ≥ 20 × 10^9^/l (continuous line) or lower (dotted line).

Median value of weekly platelet count was 79,5 × 10^9^/l (range 18–441) in patients with a BPC ≥ 20 × 10^9^/l and 40,5 × 10^9^/l (range 3–488) in those with a lower one (p=0,002). Figure 1 shows the different rise of median platelet count between the groups. Two counts > 400 × 10^9^/l occurred, one in each group and both in a splenectomized patient after the first administration of romiplostim. In these patients the platelet count was further assessed every two days until was < 200 × 10^9^/l. The patient with a higher baseline received the second injection six days later on 127 × 10^9^/l platelets, while the other received it four days later on 15 × 10^9^/l platelets. Median number of weekly platelet counts < 50 × 10^9^/l was 1 (range 0–2) in patients with a BPC ≥ 20 × 10^9^/l and 2 (range 0–4) in the other group (p=0,054). Finally, median dosages of romiplostim were 1 mcg/kg (range 0–3) and 2 mcg/kg (range 0–4) in patients with BPC ≥ 20 × 10^9^/l or lower respectively (p=0,010).

## Discussion

Time to response to romiplostim in ITP is considerably variable in both splenectomized patients and not ones. Though it is influenced by the dose and the splenectomy status, prompt responses may occur even with low dosages and in splenectomized subjects.^4^,^6^,^7^ The usual starting dose may be too small when a rapid increase in platelet count is needed.^5^ Indeed, in phase III clinical trials the dose of romiplostim was increased by 2 mcg/kg/week if platelet count was < 10 × 10^9^/l after the first dose.^4^ Moreover, in the study of romiplostim versus standard of care the starting dose was 3 mcg/kg.^8^ The necessity of a prompt response does not imply a less sensitivity to the drug. Thus, predictors of such response might avoid higher starting dosages in patients who are likely to achieve it with standard ones. In our study, patients with a BPC ≥ 20 × 10^9^/l had a median count > 50 × 10^9^/l and significantly higher than the other group during the first month. Moreover, they required a significant lower dosage. These data support our hypothesis but can be interpreted in two ways. Indeed, they might be due either to the different gap from the BPC to the threshold of response or to a different initial sensitivity to romiplostim. Two observations suggest the latter mechanism. First, the rise of median platelet count was more pronounced in patients with a higher baseline (Figure 1). The second observation is related to the counts > 400 × 10^9^/l after the first injection. The patient with a higher baseline had a progressive decrease in platelet count, while the other had a drop that led to 15 × 10^9^/l platelets in four days. The initial responses were very probably due to the absence of the spleen, but only the patient with a higher baseline maintained a safe platelet count up to the administration of the second dose. These observations are consistent with the hypothesis of a different initial sensitivity to romiplostim between the two groups, but further studies are needed to definitively solve such issue. Finally, patients with a lower baseline had a higher trend and a broader range of counts < 50 × 10^9^/l. Interestingly, both groups experienced a decrease of median platelet count concurrent with the tapering of steroids, which was less pronounced in patients with a lower baseline (Figure 1). It can be explained considering that before the decrease only 4/8 patients with lower baseline had ≥ 50 × 10^9^/l platelets while 8/10 with a higher baseline did. Consequently, half of patients with a lower baseline maintained the steroid and increased the dose of romiplostim while only 2/10 patients with a higher baseline did. Our work has important limitations, because of its retrospective and single-institution nature with a small sample size. Further, though the number of splenectomized subjects is similar between the groups, their percentage is higher in patients with a BPC < 20 × 10^9^/l.

## Conclusions

Although we cannot provide definitive conclusions, our work points out that predictors of a prompt response to romiplostim could help to individualize the treatment strategies. BPC probably reflects other features affecting the response to romiplostim, nevertheless it can be easily assessed and requires no additional costs. Thus, in our opinion its role in such setting should be further investigated. Well-designed multicenter prospective trials are needed to assess whether it can be used in clinical practice.

## Figures and Tables

**Table 1:** Baseline characteristics of the groups.

	Baseline platelet count ≥ 20 × 10^9^/1 (n=10)	Baseline platelet count < 20 × 10^9^/1 (n=8)
**Median age in years (range)**	**68 (56–85)**	**70(57–91)**
**Median weight in kilograms (range)**	74 (66–110)	72,5 (58–94)
**% of splenectomized (number/total subjects)**	20 (2/10)	37,5 (3/8)
**Median platelet count at baseline** × **10^9^/1 (range)**	25 (21–43)	10(2–16)
**Median previous treatments (range)**	3 (2–5)	3 (2–6)
**Median time in years between ITP diagnosis and romiplostim administration (range)**	3,9 (2,1–7,8)	4,3 (1,3–9,7)

**Table 2:** Percentages of patients treated with different previous therapies other than splenectomy.

	Baseline platelet count ≥ 20 × 10^9^/1 (n=10)	Baseline platelet count < 20 × 10^9^/1 (n=8)
**Steroids (number/total patients)**	100 (10/10)	100 (8/8)
**IVIG (number/total patients)**	100 (10/10)	100 (8/8)
**Rituximab (number/total patients)**	70 (7/10)	75 (6/8)
**Azathioprine (number/total patients)**	20 (2/10)	12,5 (1/8)
**Cyclosporine (number/total patients)**	20 (2/10)	25 (2/8)
**Danazole (number/total patients)**	10 (1/10)	0 (0/8)
**Other (number/total patients)**	10/(1/10)	12,5(1/8)
